# Epidemiology of *Shigella* species and serotypes in children: a retrospective substudy of the MAL-ED observational birth cohort study

**DOI:** 10.1016/j.lanmic.2024.101064

**Published:** 2025-06

**Authors:** Elizabeth T Rogawski McQuade, Jie Liu, Mustafa Mahfuz, Alexandre Havt, Tintu Varghese, Jasmin Shrestha, Furqan Kabir, Pablo Peñataro Yori, Amidou Samie, Queen Saidi, Adil Kalam, Fatima Aziz, Sahrish Muneer, Rashidul Haque, Aldo A M Lima, Maheswari Kalaivanan, Sanjaya Shrestha, Najeeha Talat Iqbal, Zulfiqar Bhutta, Margaret N Kosek, Pascal Bessong, Estomih Mduma, James A Platts-Mills, Eric R Houpt

**Affiliations:** a Department of Epidemiology, Emory University Rollins School of Public Health, Atlanta, GA, USA; b School of Public Health, Qingdao University, Qingdao, China; c Division of Infectious Diseases and International Health, University of Virginia School of Medicine, Charlottesville, VA, USA; d International Centre for Diarrhoeal Disease Research, Bangladesh, Dhaka, Bangladesh; e Federal University of Ceara, Fortaleza, Brazil; f Christian Medical College, Vellore, India; g Walter Reed/AFRIMS Research Unit Nepal, Kathmandu, Nepal; h Infectious Diseases Research Laboratory, Department Of Paediatrics and Child Health, Aga Khan University, Karachi, Pakistan; i Aga Khan University, Karachi, Pakistan; j Asociacion Benefica PRISMA, Iquitos, Peru; k University of Venda, Thohoyandou, South Africa; l Haydom Global Health Research Centre, Haydom Lutheran Hospital, Haydom, Tanzania

## Abstract

**Background:**

Shigellosis is a leading cause of diarrhoea in children globally. We aimed to assess the burden of specific *Shigella* species and *Shigella flexneri* serotypes, characterise their clinical syndromes and natural immunity, and evaluate their relevance as causes of diarrhoea and linear growth faltering.

**Methods:**

In the MAL-ED birth cohort study, children younger than 17 days were enrolled from Nov 3, 2009, to Feb 29, 2012, in Bangladesh, Brazil, India, Nepal, Pakistan, Peru, Tanzania, and South Africa and were followed up for 2 years. In this retrospective substudy, we retested *Shigella* quantitative PCR-positive diarrhoeal and non-diarrhoeal stool samples with molecular subtyping assays. We estimated the prevalence of specific *Shigella* species and serotypes, estimated their associations with diarrhoea and clinical characteristics using generalised linear mixed models, estimated their associations with linear growth using linear regression, and used longitudinal infection data to estimate protection due to previous infection using the Andersen and Gill extension of the Cox model.

**Findings:**

1715 children (874 [51·0%] male and 841 [49·0%] female) with complete follow-up to 2 years provided 45 835 stool samples collected between age 0 and 2 years. 1294 (75·5%) children had at least one *Shigella* detection in a non-diarrhoeal or asymptomatic stool and 507 (29·6%) had at least one *Shigella*-attributed diarrhoea episode. In this substudy, we were able to type 1202 samples. The most common species and serotypes were *Shigella sonnei* (366 [30·4%] of 1202)*, S flexneri* 2a (250 [20·8%]), and *S flexneri* 6 (296 [24·6%]). The associations of *S flexneri* and *S sonnei* detection with diarrhoea versus asymptomatic control stools were similar. Compared with diarrhoea episodes attributable to *S flexneri*, those attributable to *S sonnei* were less likely to be bloody (prevalence ratio 0·36 [95% CI 0·23 to 0·56]) or severe (prevalence ratio 0·58 [0·35 to 0·96]). The associations between asymptomatic *Shigella* infections and impaired linear growth at age 2 years were stronger for *S flexneri* than *S sonnei* (*S flexneri* length-for-age Z score difference –0·18 [95% CI –0·29 to –0·07] and *S sonnei* length-for-age Z score difference –0·07 [–0·21 to 0·07]). Examination of longitudinal infections showed that previous *S sonnei* infection was associated with a lower hazard of subsequent *S sonnei* diarrhoea (calibrated hazard ratio 0·41 [95% CI 0·19 to 0·90]). Otherwise, evidence for homotypic or heterotypic natural immunity was not apparent.

**Interpretation:**

This study provides data on prevailing *Shigella* species and serotypes in settings with a high burden of disease, underscoring the importance of asymptomatic *S flexneri* infection on growth impairment and the severity of *S flexneri* diarrhoea. Upcoming *Shigella* vaccines might need to induce immune responses that improve upon those produced by natural infection.

**Funding:**

Bill & Melinda Gates Foundation.

## Introduction

*Shigella* is a leading cause of childhood diarrhoea,^1^, ^2^, ^3^ with an incidence that peaks in the second year of life.^1^^,^^3^ It is the most common cause of dysentery, causes a large burden of watery diarrhoea, and leads to approximately 60 000 deaths per year.^1^^,^^4^ Subclinical or asymptomatic *Shigella* infections have been associated with poor linear growth during the first 2 years of life.^5^^,^^6^ Because asymptomatic infections are rarely diagnosed, the species and serotype distribution of these *Shigella* infections are poorly characterised.

**Research in context**

**Evidence before this study**

*Shigella* is widely recognised as a leading cause of childhood diarrhoea worldwide, but asymptomatic *Shigella* infections are also important as a contributor to poor childhood growth and as a reservoir for transmission. However, the distribution of asymptomatic *Shigella* species and serotypes are relatively unstudied. We searched PubMed from database inception to Dec 5, 2024, for studies published in any language using the search terms (“*Shigella*” or “shigellosis”) AND (“asymptomatic” or “subclinical” or “carriage”). This search yielded 304 publications; however, only four reported *Shigella* species and serotype information: two from Bangladesh, one from India, and one from Hungary. All studies used bacterial culture. This paucity of data is partly because *Shigella* diagnosis is rarely sought in asymptomatic individuals, and also because *Shigella* subtyping requires sophisticated laboratory methods. Development of an effective *Shigella* vaccine will require accurate subtype information because most vaccine candidates are specific to species and serotype.

**Added value of this study**

This study uses innovative molecular diagnostics to ascertain *Shigella* species and serotypes from a large, multisite, longitudinal birth cohort of 1715 children from eight countries, specifically 698 diarrhoeal stool specimens and 1630 asymptomatic stool specimens. These data allow for a rigorous analysis of the effect of particular species and serotypes on linear growth, on diarrhoea severity and clinical features, and on natural immunity.

**Implications of all the available evidence**

Our results suggest that subclinical infection with *Shigella flexneri*, in particular, is a major contributor to growth impairment. Our results reiterate how *S flexneri* diarrhoea is often more severe and dysenteric than *Shigella sonnei* diarrhoea. This study estimates that the leading *Shigella* vaccine candidates that target *S flexneri* 2a, 3a, and 6, and *S sonnei* could directly protect against about 80% of circulating *Shigella*. However, vaccines might need to induce strong immune responses, because we did not detect convincing evidence for serotype-specific natural immunity.

There are four species of *Shigella*, each comprised of serotypes and subserotypes based on components of the lipopolysaccharide O antigen.^7^ *Shigella flexneri*, which consists of at least 15 serotypes, and *Shigella sonnei,* which has a single serotype, are responsible for most disease.^7^^,^^8^ Other species are *Shigella dysenteriae* and *Shigella boydii.* In large, multisite studies,^8^, ^9^, ^10^ *S flexneri* has accounted for approximately two-thirds and *S sonnei* one-quarter of *Shigella* diarrhoea cases. The majority of these diarrhoeal *S flexneri* isolates were serotypes 2a, 6, 3a, 2b, and 1b.^8^, ^9^, ^10^ By contrast, the identity of which *Shigella* species or serotypes most contribute to poor childhood growth remains unclear. As current vaccines include a small number of species or serotypes, identifying prevailing *Shigella* species and serotypes among children in low-income and middle-income countries (LMICs) is important for this vaccine target population.^11^

In 2018, our group published a large and thorough multisite, longitudinal birth cohort study, Malnutrition and Enteric Disease (MAL-ED), which documented the effect of asymptomatic *Shigella* infections on poor childhood growth.^12^ This and many other studies have not included *Shigella* species or serotype information because this requires substantial laboratory resources.^1^^,^^6^^,^^12^^,^^13^ We aimed to apply molecular assays for *S sonnei* and 14 *S flexneri* serotypes to reanalyse samples that were previously PCR positive for *Shigella* in the MAL-ED study.

## Methods

### Study design and participants

The MAL-ED birth cohort study has been described previously.^14^ Briefly, from Nov 3, 2009, to Feb 29, 2012, children younger than 17 days were enrolled at eight sites: Dhaka, Bangladesh; Fortaleza, Brazil; Vellore, India; Bhaktapur, Nepal; Naushero Feroze, Pakistan; Loreto, Peru; Venda, South Africa; and Haydom, Tanzania. Children were excluded if they were very low birthweight (<1500 g), very ill, non-singleton, or the mother was younger than 16 years. Sex data were recorded based on information confirmed by the caregiver.

Fieldworkers made biweekly home visits until participants were aged 2 years to identify diarrhoea episodes, defined as three or more loose or watery stools in 24 h or one stool with visible blood. Symptoms and severity were collected for each episode. Height and weight were measured monthly and at age 5 years and converted to Z scores based on WHO child growth standards.^15^ Stool samples were collected during diarrhoea and monthly. Myeloperoxidase (log ng/mL), a marker of neutrophil activation, was measured by ELISA (ALPCO, Salem, NH, USA) in non-diarrhoeal and asymptomatic stools collected monthly in the first year of life and quarterly in the second year.^16^^,^^17^

Ethics approval was obtained from each site’s institutional review board and caregivers provided written informed consent, including for additional laboratory testing and analysis of collected samples as conducted for this additional study.

### Procedures

In children with complete follow-up to age 2 years, stool samples were previously tested for enteropathogens by quantitative PCR (qPCR) using custom-designed TaqMan Array Cards (ThermoFisher Scientific, Carlsbad, CA, USA). *Shigella* and enteroinvasive *Escherichia coli* were detected using the *ipaH* gene. For this retrospective substudy, all samples with *ipaH* cycle threshold (Ct) less than 30 that were available in sufficient quantity were retested with published *Shigella* species and serotyping assays by multiplex real-time PCR (rtPCR; appendix p 2).^13^ Retesting was completed on stored DNA at all sites except Brazil, Nepal, and Tanzania, where testing was instead completed using DNA re-extracted from whole stool (appendix p 2). To accommodate different rtPCR platforms at different sites, three PCR panels were formulated, including eight *S flexneri* specific targets for serotype identification, an *S sonnei* assay, *ipaH* to reconfirm *Shigella* detection, and an external control (phocine herpesvirus; appendix p 2). *S flexneri* serotypes were assigned as previously described (appendix p 2).^13^ As previously, we required the Ct of the *S flexneri* serotyping target or the *S sonnei* target to fall within 5 Cts of the *ipaH* Ct. When two or more targets were required to detect a serotype, the Ct difference between the two or more targets had to be within 2. Samples were classified as *S flexneri* if any of the *S flexneri* serotypes were identified.

### Statistical analysis

The aim was to enrol 200 children per site in the parent MAL-ED study. For the current analysis, we used all available samples that met the inclusion criteria and no additional sample size calculations were performed.

For each analysis, confounding variables were identified by causal directed acyclic graphs. In the context of mixed infections, associations for specific *Shigella* species or serotypes were assumed to be independent and analyses were adjusted for the presence of other species or serotypes. Species and serotypes in leading vaccine candidates included *S flexneri* 1b, 2a, 3a, and 6 and *S sonnei*. To associate *Shigella* species and serotypes with diarrhoea, we used quantity-specific and pathogen-specific attributable fractions.^1^ A generalised linear mixed-effects logistic regression model (GLMM) was fit for each *Shigella* species, where the outcome was diarrhoea and predictors were linear and quadratic terms for pathogen quantity (Ct), sex, test batch, linear and quadratic terms for child age, a random slope for site, and a random intercept for each individual. Random effects terms were assumed to be independent. Choice of specification for quantitative predictors was determined by model fit per Akaike information criterion.

In sensitivity analysis we repeated estimation of associations as risk ratios using a Poisson GLMM to approximate log-binomial regression. To attribute *Shigella* species as the cause of diarrhoea, we calculated an episode-specific attributable fraction (AF_e_), as AF_e_ = 1 – 1/OR_e_, where OR_e_ is the species-specific and quantity-specific odds ratio from the GLMM. To estimate the associations between pathogen-attributable diarrhoea and clinical characteristics, including blood in stool, fever, diarrhoea duration, dehydration, vomiting, and stool frequency, a Poisson GLMM was fit for each characteristic and predictors included the AF_e_ for each pathogen, age, sex, and nested random effects for site and individual. Coefficients for the prevalence ratio and 95% CI were scaled to the AF_e_ range for each pathogen. Severe diarrhoea was defined as a score greater than 6 as previously described.^18^

We estimated associations (mean differences and 95% CI) of asymptomatic *S flexneri, S sonnei*¸ and *Shigella* of uncertain type with length-for-age Z score at age 2 years and height-for-age Z score at age 5 years using linear regression adjusted for site, enrolment length-for-age Z score, sex, socioeconomic status,^19^ exclusive breastfeeding in the first 6 months of life, maternal height, and burden of other pathogens.^6^ *Shigella* exposures were dichotomised to one or more detection of the species or serotype versus none because sample size precluded higher resolution comparisons. Estimates were stratified by site.

We associated one or more species-specific *Shigella*-attributable diarrhoea episodes with length-for-age Z score at 2 years and height-for-age Z score at 5 years using linear regression (mean difference and 95% CI), adjusted for enrolment length-for-age Z score, sex, socioeconomic status, exclusive breastfeeding in the first 6 months of life, maternal height, number of diarrhoea episodes attributable to the nine most attributable enteropathogens, number of non-attributable diarrhoea episodes, and number of episodes treated with antibiotics. We associated each species-specific *Shigella*-attributable diarrhoea episode with length-for-age Z score 3 months after the episode using linear regression with generalised estimating equations to account for clustering of episodes within children and the same adjustments using an exchangeable working correlation matrix and robust variance.

We estimated associations (mean difference and 95% CI) of species-specific *Shigella* detections and quantity with myeloperoxidase concentration in the same stool using linear regression models with generalised estimating equations. *Shigella* quantity was based on the original *ipaH* Ct and was modelled in tertiles and continuously per log_10_ increase in quantity. Models were adjusted for site, age, sex, and stool consistency.

We estimated homotypic and heterotypic natural immunity as the effects of previous species-specific *Shigella* infections on the hazard of subsequent asymptomatic infections and diarrhoea.^12^ Asymptomatic infections and diarrhoea were defined at least 21 days after previous infection,^12^ and required detection of *ipaH* at quantification cycle less than or equal to 30·507 (ie, the quantity associated with diarrhoea) and identification of *Shigella* species or serotypes. Subsequent diarrhoea episodes had to be further accompanied by symptoms. We used the Andersen and Gill extension of the Cox model for recurrent events with robust standard errors. Children contributed multiple risk periods defined by birth or age 21 days after a previous infection to age at subsequent outcome or end of the study at age 2 years. We estimated hazard ratios (HRs) with 95% CIs, which compared children who had one or more previous asymptomatic or diarrhoea infection with children who had no previous infections. HRs less than 1 provide evidence of natural immunity. Models were adjusted for site, sex, socioeconomic status, enrolment weight-for-age Z score, maternal height, maternal education, crowding in the home, and exclusive breastfeeding. In sensitivity analyses, we stratified estimates by the first and second year of life, further adjusted for any antibiotic use and macrolide or fluoroquinolone use, and defined the exposure variable only by species-specific or serotype-specific previous diarrhoea episodes (ie, excluding infections as a marker of previous exposure).

We calibrated estimates with negative control associations between *Shigella* outcomes and previous exposure to ten other bacteria using the EmpiricalCalibration package in R.^20^ We generated a systematic error model for each *Shigella* outcome that fit a Gaussian distribution to the negative control estimates and accounted for the sampling error of each estimate. Calibrated association estimates and CIs for the effect of homotypic immunity were generated based on percentiles of a calibrated distribution that incorporated random error and the systematic error model.^20^

Statistical analyses were conducted in R (version 4.1.0).

### Role of the funding source

The funder of the study had no role in study design, data collection, data analysis, data interpretation, or writing of the report.

## Results

1715 children (874 [51·0%] male and 841 [49·0%] female) with complete follow-up to 2 years provided 45 835 stool samples collected between age 0 and 2 years. 1294 (75·5%) children had at least one *Shigella* detection in a non-diarrhoeal or asymptomatic stool and 507 (29·6%) had at least one *Shigella*-attributed diarrhoea episode, of whom 160 (31·6%) had at least one subsequent *Shigella* diarrhoea episode. Of 2872 stool samples that tested positive for *ipaH* with a Ct less than 30 in the original qPCR analysis, 2328 (81·1%) were available in sufficient quantity and were retested using the *Shigella* species and serotyping assays. Specimen availability varied by site, with a range of 60–100% of samples retested across sites. Of 2328 retested samples, 698 (30·0%) were diarrhoeal stools and 1630 (70·0%) were non-diarrhoeal or asymptomatic stools collected during monthly surveillance (appendix p 3). Among 2204 retested stools that were *ipaH* positive on retest, 1202 (54·5%) could have a species or *S flexneri* serotype identified; 124 (5·3%) of 2328 retested samples were *ipaH* negative on retest. *ipaH* Ct was a mean of 2·5 (95% CI 1·41–3·66) higher on retest compared with the original analysis, and this was consistent whether the retest was done on newly extracted DNA or stored DNA. The distribution of *ipaH* Ct values was not substantially different across species or *S flexneri* serotypes or untyped samples (appendix p 4).

Among 1202 typed samples (of which 354 from 281 children were diarrhoeal and 848 from 547 children were from non-diarrhoeal or asymptomatic cases), the most common species and serotypes were *S sonnei* (366 [30·4%])*, S flexneri* 2a (250 [20·8%]), and *S flexneri* 6 (296 [24·6%] table 1). *S flexneri* was more common than *S sonnei* in all sites except Brazil. The most common *S flexneri* serotype was *S flexneri* 6 in all sites except in Brazil and Pakistan, where *S flexneri* 2a was more common. *Shigella* vaccine candidates under development that target particular *Shigella* combination species and serotypes would provide direct coverage for 50·7–80·4% of typed samples depending on the antigens included in the vaccine (table 1). Prevalence of species and serotypes among all samples that had any detectable *ipaH* on retest are shown in the appendix (p 5).

**Table 1:** *Shigella sonnei* and *Shigella flexneri* serotype distribution across stool samples

	Site								Stool type		All (n=1202)
	Bangladesh (n=190)	Brazil (n=22)	India (n=173)	Nepal (n=144)	Peru (n=266)	Pakistan (n=98)	South Africa (n=22)	Tanzania (n=287)	Diarrhoeal (n=354)	Non-diarrhoeal or asymptomatic (n=848)	
*S flexneri*	118 (62·1%)	7 (31·8%)	116 (67·1%)	81 (56·3%)	213 (80·1%)	72 (73·5%)	17 (77·3%)	240 (83·6%)	219 (61·9%)	645 (76·1%)	864 (71·9%)
*S flexneri* 1a	4 (2·1%)	0	7 (4·0%)	2 (1·4%)	5 (1·9%)	15 (15·3%)	0	7 (2·4%)	8 (2·3%)	32 (3·8%)	40 (3·3%)
*S flexneri* 1b	0	0	4 (2·3%)	3 (2·1%)	3 (1·1%)	4 (4·1%)	1 (4·5%)	8 (2·8%)	8 (2·3%)	15 (1·8%)	23 (1·9%)
*S flexneri* 1d	0	0	0	0	1 (0·4%)	0	0	2 (0·7%)	1 (0·3%)	2 (0·2%)	3 (0·2%)
*S flexneri* 2a	41 (21·6%)	5 (22·7%)	39 (22·5%)	16 (11·1%)	68 (25·6%)	21 (21·4%)	5 (22·7%)	55 (19·2%)	80 (22·6%)	170 (20·0%)	250 (20·8%)
*S flexneri* 2b	2 (1·1%)	1 (4·5%)	1 (0·6%)	1 (0·7%)	0	3 (3·1%)	0	17 (5·9%)	4 (1·1%)	21 (2·5%)	25 (2·1%)
*S flexneri* 3a	14 (7·4%)	0	2 (1·2%)	8 (5·6%)	20 (7·5%)	4 (4·1%)	2 (9·1%)	35 (12·2%)	23 (6·5%)	62 (7·3%)	85 (7·1%)
*S flexneri* 3b	1 (0·5%)	0	6 (3·5%)	1 (0·7%)	10 (3·8%)	7 (7·1%)	0	10 (3·5%)	10 (2·8%)	25 (2·9%)	35 (2·9%)
*S flexneri* 4a	8 (4·2%)	1 (4·5%)	9 (5·2%)	6 (4·2%)	8 (3·0%)	3 (3·1%)	0	5 (1·7%)	13 (3·7%)	27 (3·2%)	40 (3·3%)
*S flexneri* 4b	1 (0·5%)	0	1 (0·6%)	1 (0·7%)	6 (2·3%)	0	0	7 (2·4%)	3 (0·8%)	13 (1·5%)	16 (1·3%)
*S flexneri* 5a	0	0	1 (0·6%)	3 (2·1%)	2 (0·8%)	0	0	7 (2·4%)	0	13 (1·5%)	13 (1·1%)
*S flexneri* 5b	0	0	1 (0·6%)	0	1 (0·4%)	0	0	1 (0·3%)	1 (0·3%)	2 (0·2%)	3 (0·2%)
*S flexneri* 6	42 (22·1%)	0	49 (28·3%)	37 (25·7%)	80 (30·1%)	16 (16·3%)	7 (31·8%)	65 (22·6%)	60 (16·9%)	236 (27·8%)	296 (24·6%)
*S flexneri* 7a	8 (4·2%)	0	0	1 (0·7%)	0	0	0	1 (0·3%)	3 (0·8%)	7 (0·8%)	10 (0·8%)
*S flexneri* X	7 (3·7%)	0	5 (2·9%)	2 (1·4%)	19 (7·1%)	5 (5·1%)	2 (9·1%)	36 (12·5%)	16 (4·5%)	60 (7·1%)	76 (6·3%)
*S sonnei*	76 (40·0%)	15 (68·2%)	62 (35·8%)	66 (45·8%)	58 (21·8%)	30 (30·6%)	5 (22·7%)	54 (18·8%)	141 (39·8%)	225 (26·5%)	366 (30·4%)
Mixed∗	14 (7·4%)	0	14 (8·1%)	3 (2·1%)	15 (5·6%)	8 (8·2%)	0	22 (7·7%)	16 (4·5%)	60 (7·1%)	76 (6·3%)
Combination of serotypes in leading vaccine candidates											
*S flexneri* 2a, 3a, or 6, or *S sonnei*	166 (87·4%)	20 (90·9%)	146 (84·4%)	125 (86·8%)	218 (82·0%)	69 (70·4%)	19 (86·4%)	203 (70·7%)	296 (83·6%)	670 (79·0%)	966 (80·4%)
*S flexneri* 1b, 2a, or 3a, or *S sonnei*	129 (67·9%)	20 (90·9%)	106 (61·3%)	93 (64·6%)	144 (54·1%)	58 (59·2%)	13 (59·1%)	151 (52·6%)	249 (70·3%)	465 (54·8%)	714 (59·4%)
*S flexneri* 2a or *S sonnei*	115 (60·5%)	20 (90·9%)	100 (57·8%)	82 (56·9%)	123 (46·2%)	50 (51·0%)	10 (45·5%)	109 (38·0%)	220 (62·1%)	389 (45·9%)	609 (50·7%)

∗ Mixed detections are counted for each individual species or serotype and for mixed.

The associations of particular species or serotypes with diarrhoea were similar for *S flexneri*, *S sonnei*, and *Shigella* of uncertain type (figure). For example, at a Ct of 25, the odds ratio (OR) for *Shigella* detection and diarrhoea was similar for *S flexneri* (OR 2·57 [95% CI 2·00–3·30]), *S sonnei* (3·35 [2·36–4·78]), and untyped *Shigella* (2·62 [2·13–3·22]). Similarly, the Ct with an OR of 2, corresponding to AF_e_ = 0·5, was similar for *S flexneri* (26·3)*, S sonnei* (28·5)*,* and untyped *Shigella* (27·0). The trends by pathogen quantity of the associations of *Shigella* species with diarrhoea were similar when estimated as risk ratios, although the magnitudes of association were smaller (appendix p 6).

**Figure:**
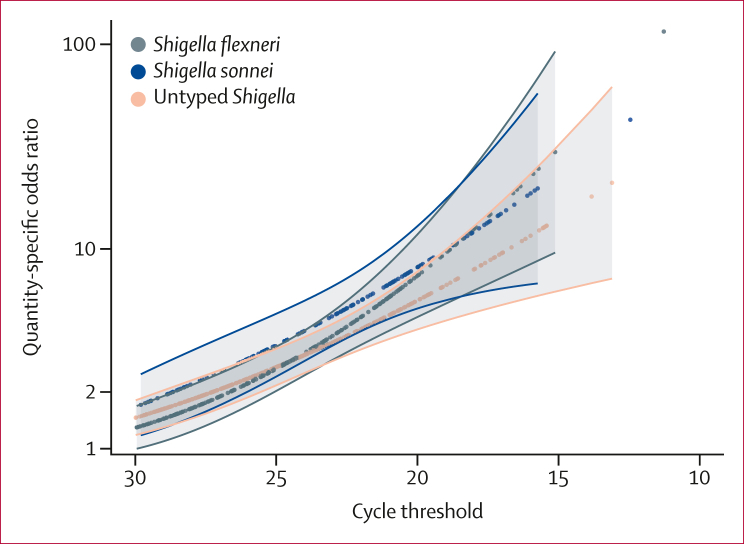
Association between *Shigella* quantity and diarrhoea by *Shigella* species Association is the species-specific and quantity-specific odds ratio from a generalised linear mixed-effects logistic regression model in which the outcome was diarrhoeal versus non-diarrhoeal stool, and predictors were linear and quadratic terms for pathogen quantity (using the cycle threshold value), child sex, TaqMan Array Card test batch, linear and quadratic terms for child age in 3-month intervals, a random slope for each site, and a random intercept for each individual. Shaded areas show 95% CIs.

Of the 698 diarrhoeal stools that were re-tested, *ipaH* was detected in 681 (97·6%) and 334 (49·0%) were typed as either *S flexneri* or *S sonnei*. Diarrhoea episodes attributable to *S sonnei* were less likely to be bloody (prevalence ratio 0·36 [95% CI 0·23–0·56]) or severe (prevalence ratio 0·58 [0·35–0·96]) than those attributable to *S flexneri* (table 2). The prevalence ratio for fever, dehydration, high stool frequency, and prolonged duration was also lower for *S sonnei* than *S flexneri* episodes, although these estimates were not statistically significant (table 2). There was no evidence for a difference in the presence of vomiting between episodes attributed to *S flexneri* and *S sonnei*. Episodes attributed to a *Shigella* of uncertain type had a lower prevalence ratio for all severity characteristics, although the associations were imprecise, with most CIs crossing 1 (table 2).

**Table 2:** Prevalence ratios for associations between diarrhoea due to specific *Shigella* species and clinical features of diarrhoea (n=681)

	Blood (n=103)	Fever (n=208)	Duration ≥7 days (n=127)	Dehydration (n=71)	Vomiting (n=126)	High frequency, more than six loose stools in 24 h (n=163)	Severe (n=77)
*Shigella flexneri*	1 (ref)	1 (ref)	1 (ref)	1 (ref)	1 (ref)	1 (ref)	1 (ref)
*Shigella sonnei*	0·36 (0·23–0·56)	0·85 (0·62–1·16)	0·76 (0·51–1·13)	0·63 (0·38–1·06)	0·97 (0·65–1·44)	0·82 (0·57–1·18)	0·58 (0·35–0·96)
*Shigella* of uncertain type	0·61 (0·37–1·01)	0·92 (0·63–1·35)	0·71 (0·43–1·18)	0·42 (0·19–0·92)	0·58 (0·33–1·03)	0·85 (0·55–1·31)	0·62 (0·33–1·18)

Data are prevalence ratio (95% CI).

The adjusted associations between asymptomatic *Shigella* infections and linear growth were stronger for *S flexneri* than for *S sonnei* (table 3). Children who had at least one *S flexneri* infection had a lower length-for-age Z score at age 2 years compared with children with no *S flexneri* infections, whereas children with at least one *S sonnei* infection had similar length-for-age Z scores at age 2 years compared with children without *S sonnei* infections (table 3). These associations persisted to age 5 years. These results were consistent across sites and time, with *S flexneri* more strongly associated with decrements in length-for-age Z score than *S sonnei* at most sites at age 2 years and in height-for-age Z score at all sites at age 5 years (appendix p 7). However, estimates for each site are less precise than the overall estimates owing to small sample sizes.

**Table 3:** Association of subclinical *Shigella* infections and *Shigella-*attributable diarrhoea episodes with length-for-age Z score at age 2 years and height-for-age Z score at age 5 years

	Participants (n=1469)	Length-for-age Z score difference∗† at age 2 years (95% CI)	Height-for-age Z score difference∗† at age 5 years (95% CI)	Length-for-age Z score difference‡ 3 months following episode (95% CI)
**One or more subclinical infection**§				
*Shigella flexneri*	395 (26·9%)	–0·18 (–0·29 to –0·07)	–0·24 (–0·36 to –0·12)	NA
*Shigella sonnei*	174 (11·8%)	–0·07 (–0·21 to 0·07)	–0·03 (–0·17 to 0·12)	NA
*Shigella* type unknown	654 (44·5%)	–0·02 (–0·11 to 0·07)	–0·08 (–0·18 to 0·02)	NA
**One or more attributable diarrhoea episode**				
*S flexneri*	125 (8·5%)	–0·14 (–0·30 to 0·02)	–0·08 (–0·26 to 0·10)	–0·05 (–0·11 to 0·02)
*S sonnei*	112 (7·6%)	–0·13 (–0·30 to 0·04)	–0·21 (–0·40 to –0·02)	–0·02 (–0·09 to 0·04)
*Shigella* type unknown	284 (19·3%)	–0·08 (–0·21 to 0·05)	–0·11 (–0·26 to 0·03)	0·04 (–0·02 to 0·10)

NA=not applicable.

∗ Subclinical infection models adjusted for site, enrolment length-for-age Z score, sex, socioeconomic status, exclusive breastfeeding in the first 6 months, maternal height, and proportion of non-diarrhoeal stools positive for *Cryptosporidium*, *Campylobacter* spp*, Giardia*, enteroaggregative *Escherichia coli*, enterotoxigenic *E coli*, typical enteropathogenic *E coli*, atypical enteropathogenic *E coli*, norovirus, adenovirus 40/41, astrovirus, sapovirus, and *Enterocytozoon bieneusi*.

† Diarrhoea models adjusted for enrolment length-for-age Z score, sex, socioeconomic status, exclusive breastfeeding in the first 6 months, maternal height, number of diarrhoea episodes attributable to the nine other enteropathogens with the highest attributable diarrhoea incidence, number of non-attributable diarrhoea episodes, and number of episodes treated with any antibiotics.

‡ Diarrhoea models adjusted for age, site, sex, socioeconomic status, maternal height, length-for-age Z score at the beginning of the interval, exclusive breastfeeding, diarrhoea episodes due to each of the nine other enteropathogens with the highest attributable diarrhoea incidence, and the number of non-attributable diarrhoea episodes in the same period.

§ Subclinical infections were defined as at least one detection with *ipaH* cycle threshold less than 30 because typing assays were only conducted among samples with *ipaH* cycle thresholds less than 30.

The associations between at least one species-specific attributable diarrhoea episode with length-for-age Z score at age 2 years were similar between *S flexneri* and *S sonnei* (table 3). *S sonnei* diarrhoea was more strongly associated with worse height-for-age Z score at age 5 years than *S flexneri* diarrhoea (table 3). Diarrhoea caused by either species was associated with small, non-significant decrements in length-for-age Z score 3 months after the episode (table 3).

The association with myeloperoxidase was similar between *S flexneri* and *S sonnei* infections (appendix p 8). Adjusting for confounding variables, *S flexneri* was associated with a 1·58 ng/mL (95% CI 1·36–1·84) higher myeloperoxidase concentration than stools without *S flexneri*. *S sonnei* was associated with a 1·77 ng/mL (1·38–2·27) higher myeloperoxidase concentration than stools without *S sonnei*. There was a similar dose response between *Shigella* quantity and myeloperoxidase concentration for both species and *Shigella* of uncertain type, such that higher quantities of *Shigella* were consistently associated with higher concentrations of myeloperoxidase (appendix p 8).

In our analysis of longitudinal infections to evaluate evidence for natural immunity, 188 (34·4%) of 547 children with a speciated or serotyped *Shigella* detection in non-diarrhoeal stools had a repeat detection of the same *Shigella* species or serotype. Time to subsequent *S sonnei* diarrhoea episode was shorter than time to subsequent *S flexneri* diarrhoea episode (appendix p 9). 40 (15·6%) of 281 children with *Shigella* diarrhoea that was speciated or serotyped had at least one subsequent *Shigella* diarrhoea episode with the same species or serotype. Previous *S sonnei* infection was associated with a 59% lower hazard (adjusted HR [aHR] 0·41 [95% CI: 0·19–0·90]) of subsequent *S sonnei* diarrhoea after adjusting for confounding variables and calibrating estimates using the negative controls; however, there was no protection against subsequent asymptomatic *S sonnei* infection (table 4). There was no evidence of homotypic immunity for *S flexneri*, such that previous infection with the same species or serotype was not associated with reduced risk of asymptomatic infection or diarrhoea with the same species or serotype, and in some cases there was an associated increase in risk (table 4). Estimates of protection in the second year of life were similar to overall, although no longer statistically significant, whereas estimates in the first year of life were very imprecise (appendix p 10). Estimates of protection were similar when additionally adjusting for antibiotic use (appendix p 11) and when defining previous exposure by previous diarrhoea instead of previous asymptomatic infection (appendix p 12).

**Table 4:** Estimates of protection against *Shigella* species-specific and serotype-specific subclinical infections and attributable diarrhoea due to one or more previous infections (asymptomatic or diarrhoea) from the same species or serotype

	Adjusted∗ HR (95% CI)	Calibrated† HR (95% CI)
***Shigella flexneri***		
Infection	2·11 (1·73–2·57)	1·74 (1·38–2·18)
Diarrhoea	1·65 (1·14–2·38)	1·29 (0·89–1·87)
***Shigella sonnei***		
Infection	2·14 (1·52–3·02)	1·63 (1·10–2·40)
Diarrhoea	0·57 (0·26–1·25)	0·41 (0·19–0·90)
***S flexneri*****2a**		
Infection	3·07 (1·93–4·90)	2·50 (1·56–4·02)
Diarrhoea	2·03 (0·90–4·56)	1·79 (0·77–4·15)
***S flexneri*****3a**		
Infection	3·82 (1·71–8·49)	2·81 (1·21–6·51)
Diarrhoea	2·25 (0·30–16·99)	1·69 (0·22–13·12)
***S flexneri*****6**		
Infection	3·24 (2·32–4·51)	2·63 (1·67–4·14)
Diarrhoea	2·31 (0·90–5·94)	1·64 (0·63–4·28)
***S flexneri*****X**		
Infection	1·71 (0·59–5·00)	1·37 (0·47–4·01)
Diarrhoea	··	··

HR=hazard ratio.

∗ HRs adjusted for site, socioeconomic status, sex, enrolment weight-for-age Z score, maternal education, maternal height, crowding, and exclusive breastfeeding in first 6 months.

† HRs adjusted for the same variables as the adjusted HRs and calibrated based on negative control estimates.

Adjusting for confounding variables, previous *S flexneri* infection was associated with a slightly lower hazard of subsequent *S sonnei* diarrhoea (aHR 0·79 [95% CI 0·44–1·42]), although the estimate was imprecise and not statistically significant. By contrast, previous *S sonnei* infection was associated with an increase in hazard of subsequent *S flexneri* diarrhoea (aHR 1·86 [1·16–2·97]). It has been predicted that some *S flexneri* serotypes might provide cross-protection against certain other serotypes via shared O antigen epitopes, yet we observed no differences in protection from previous *S flexneri* 2a or 3a infection against diarrhoea due to *S flexneri* types with cross-protective antigens compared with types that are not expected to be cross protective (appendix p 13).^21^ All estimates of cross protection were non-significant with wide 95% CIs (data not shown).

## Discussion

In this analysis of 1202 *Shigella* infections, *S flexneri* 2a and 6 and *S sonnei* were the most common species and serotypes identified among children in low-income and middle-income countries. The prevalence of these species and serotypes was generally expected,^8^^,^^10^ although we observed a higher prevalence of *S flexneri* 3b and X than did the large GEMS^8^ and VIDA studies,^9^ and we observed less *S flexneri* 1b and 3a than did a large study from Asia.^10^ Approximately 80% of typed infections were *S sonnei* or *S flexneri* 2a, 3a, or 6, the components of several leading vaccine candidates.^11^

Perhaps our most important finding was that asymptomatic *S flexneri* infections were uniquely associated with decrements in linear growth, a finding consistent across sites and persistent to age 5 years. The mechanism for this effect on linear growth is uncertain. One might hypothesise that *S flexneri* is more invasive than *S sonnei* even when asymptomatic. However, both species had similar effects on systemic inflammation as measured by myeloperoxidase. Consistent with previous studies,^10^^,^^22^, ^23^, ^24^ *S flexneri* diarrhoea was more likely to be dysenteric than *S sonnei* and more likely to be severe. *S flexneri* and *S sonnei* were similarly attributed as the cause of diarrhoea based on the quantity of pathogen detected, suggesting a single quantitative *ipaH* Ct cutoff to assign diarrhoea aetiology is sufficient.

Evidence for natural immunity to particular *Shigella* species, and lack thereof, was surprising. We found that previous *S sonnei* infection conferred protection against subsequent *S sonnei* diarrhoea; however, we did not observe protection against subsequent *S sonnei* carriage. The absence of protection against carriage has also been noted previously.^25^ We observed no protection from *S flexneri* against either subsequent disease or carriage, contrasting with the prevailing view that *Shigella* infections provide homotypic protection.^21^^,^^26^^,^^27^ For example, in animal models, *S flexneri* 2a infection provides protection against rechallenge.^28^ Likewise, human data on *Shigella* natural immunity from a Chilean cohort showed protection against illness with *S sonnei*, *S flexneri* 2a, and *S flexneri* 6.^29^ In that smaller study, only ten (10·8%) of 93 participants with shigellosis had a repeat episode, whereas in this study, 160 (31·6%) of 507 participants with shigellosis had a repeat episode.

On the contrary, previous infections of most serotypes were associated with increased risk of diarrhoea due to the same serotype. We posit several possible explanations. First, natural immunity to these *S flexneri* serotypes might be truly limited or absent in these low-income and middle-income country settings, for instance due to a high force of infection. Second, these children were younger than 2 years, and one-third had stunting, which could hamper an adequate immune response. Alternatively, natural immunity to *S flexneri* serotypes could still exist, but limitations in study design obscured it. For instance, children exposed to *Shigella* might be at higher risk of being re-exposed, and the negative control associations might not completely capture this bias. Despite requiring 21 days between infections and a high quantity of pathogen present, subsequent asymptomatic detections could represent prolonged carriage rather than re-infections. Nonetheless, our results are consistent with a previous analysis of natural immunity seen in this cohort, in which the estimate of protection from any previous *Shigella* infection against subsequent *Shigella* diarrhoea was 21% (95% CI 5–35),^12^ approximately the average of our estimates for *S flexneri* (–29%) and *S sonnei* (59%).

This study was limited by the inability to identify species or serotypes for almost half of the samples that were retested. Therefore, several of our estimates were affected by substantial uncertainty given the limited sample size in some analyses. Furthermore, misclassification might have biased analyses of linear growth faltering and natural immunity. Nonetheless, despite the average yield of molecular serotyping, the number of serotyped samples (n=1202) was still substantial, particularly given the low sensitivity of culture (only 0·9% of tested samples were *Shigella-*culture positive). The analysis was limited by the potential for unmeasured and residual confounding. We also did not adjust for other possible causes of growth faltering. Myeloperoxidase was measured only in non-diarrhoeal stools. Finally, the estimation of natural immunity might also have been affected by the built-in selection bias of HRs.

In conclusion, we found that both *S flexneri* and *S sonnei* are important causes of diarrhoea among children in low-resource settings; however, *S flexneri* appeared to be more detrimental to long-term child growth. Evidence for natural immunity to *S sonnei* is encouraging for vaccine development.^30^ The explanation for the absence of natural immunity seen with *S flexneri* infections is unclear and merits careful assessment in future studies and vaccine trials, because an effective *S flexneri* vaccine is an important global health priority.

## Data sharing

Most data collected and analysed in this study are included within the manuscript or in the appendices. The remaining datasets can be made available on reasonable request to the corresponding author.

## Declaration of interests

We declare no competing interests.
