# Supplementary material for: Epidemiology of Shigella species and serotypes in children: a retrospective substudy of the MAL-ED observational birth cohort study
Source: Lancet Microbe. 2025 Jun;6(6):None. doi: 10.1016/j.lanmic.2024.101064 (PMC12134051; doi:10.1016/j.lanmic.2024.101064)
Supplement: Supplementary appendix [file mmc1.pdf]

THE LANCET
Microbe

Supplementary appendix

This appendix formed part of the original submission and has been peer reviewed. We post it as supplied by the authors.

Supplement to: Rogawski McQuade ET, Liu J, Mahfuz M, et al. Epidemiology of *Shigella* species and serotypes in children: a retrospective substudy of the MAL-ED observational birth cohort study. *Lancet Microbe* 2025. https://doi.org/10.1016/j.lanmic.2024.101064

Epidemiology of *Shigella* species and serotypes in children: results from the MAL-ED study

Supplemental appendix

Contents

Methods.. 2

Table S1. The composition of qPCR panels and the corresponding *Shigella* species and *S. flexneri* serotypes. 2

Table S2. The primer and probe sequences for the qPCR assays. 3

Results.. 3

Table S3. Number and type of samples by site among 2328 samples that were retested with the *Shigella* typing assays in the MAL-ED study. 3

Figure S1. Distribution of *ipaH* Cts by *Shigella* species detected at each site. 4

Table S4. *S. sonnei* and *S. flexneri* serotype distribution across sites among 2295 samples that had detectable *ipaH* on retest with the *Shigella* typing assays in the MAL-ED study. 5

Figure S2. Risk ratio between *Shigella* quantity and diarrhoea by *Shigella* species in MAL-ED cohort. 6

Table S5. Site-specific associations of subclinical *Shigella* infections with length-for-age z-score (LAZ) at two years of age and height-for-age z-score (HAZ) at five years of age in the MAL-ED cohort. 7

Table S6. Association of *Shigella* species with myeloperoxidase concentration in the same stool sample. 8

Figure S3. Dose response between quantity of specific *Shigella* species detected with myeloperoxidase concentration in the same stool sample. 8

Table S7. Median time to subsequent outcome (infection or diarrhoea) following a prior infection by *Shigella* species or serotype. 9

Table S8. Estimated of protection against *Shigella* species/serotype specific subclinical infections and attributable diarrhoea due to 1 or more prior infections (asymptomatic or diarrhoea) from the same species/serotype in the MAL-ED cohort in the first (0-11 months) and second (12-23 months) years of life. 10

Table S9. Estimates of protection against *Shigella* species/serotype specific subclinical infections and attributable diarrhoea due to 1 or more prior infections (asymptomatic or diarrhoea) from the same species/serotype in the MAL-ED cohort, additionally adjusting for antibiotic use. 11

Table S10. Estimates of protection against *Shigella* species/serotype specific attributable diarrhoea due to 1 or more prior diarrhoea episodes from the same species/serotype in the MAL-ED cohort. 12

Table S11. Expressions of cross protection from *S. flexneri* 2a and *S. flexneri* 3a against other *S. flexneri* serotypes and *S. sonnei*.[2] 13

References.. 13

Methods

***Shigella* serotyping methods.** Nucleic acid was extracted from stool samples with QIAamp Fast DNA Stool mini kit (Qiagen, Hilden, Germany), and multiplex qPCR reactions were performed with AgPath One Step Real time PCR reagents (ThermoFisher Scientific, Carlsbad, California, USA) as previously described.[1] The cycling conditions were set as reverse transcription at 45°C for 20 min, 40 cycles of 95°C for 15 sec, 60°C for 1min. The qPCR primers and probes (Table S2) were synthesized by Integrated DNA Technologies (IDT, Coralville, Iowa, USA). External control (Phocine herpesvirus, PhHV) was spiked into each sample during nucleic acid extraction to monitor the extraction and amplification efficiency. One extraction blank was incorporated per batch of extraction to rule out lab contamination. One no-template control (nuclease free water) and one pooled synthetic positive control were included per PCR run to validate the results. The assay thresholds were set based on the positive controls to ensure consistency across instruments including QuantStudio 7 Flex (ThermoFisher Scientific, Carlsbad, California, USA) used in the laboratories in India and Peru, ViiA 7 (ThermoFisher Scientific, Carlsbad, California, USA) used in the laboratory in Tanzania, QuantStudio 5 (ThermoFisher Scientific, Carlsbad, California, USA) used in the laboratories in Brazil and South Africa, ABI Fast 7500 (ThermoFisher Scientific, Carlsbad, California, USA) used in the laboratory in Nepal, and BioRad CFX 96 (BioRad, Hercules, California, USA) used in the laboratories in Bangladesh and Pakistan. *S. sonnei* or *S. flexneri* serotypes were determined (Table S1 below) when the results of the species or serotype specific targets satisfied two criteria:

1) The Ct difference between *ipaH* and specific target was less than 5
2) When multiple targets were required to assign one *S. flexneri* serotype, the Ct difference between targets was less than 2.

Table S1. **The composition of qPCR panels and the corresponding *Shigella* species and *S. flexneri* serotypes.** The colours indicate the fluorophore of the probes, blue for FAM, green for VIC, red for Texas Red, and purple for Cy5.

***Shigella* species or *S. flexneri* serotype**	**qPCR panel I**	**qPCR panel II**	**qPCR panel III**
***S. flexneri* 1a**		*gtrI*	
***S. flexneri* 1b**	*oac*	*gtrI*	
***S. flexneri* 1d**	*gtrX*	*gtrI*	
***S. flexneri* 2a**	*gtrII*		
***S. flexneri* 2b**	*gtrII*, *gtrX*		
***S. flexneri* 3a**	*oac*, *gtrX*		
***S. flexneri* 3b**	*oac*		
***S. flexneri* 4a**		*gtrIV*	
***S. flexneri* 4b**	*oac*	*gtrIV*	
***S. flexneri* 5a**	*oac*		*gtrV*
***S. flexneri* 5b**	*oac*, *gtrX*		*gtrV*
***S. flexneri* 6**	*wzx6*		
***S. flexneri* 7a**		*gtrI*	*gtrIc*
***S. flexneri* X**	*gtrX*		
S. sonnei		*Rhs, pm*	
Shigella		*ipaH*	*ipaH3*
**External control (PhHV)**			*gB*

Table S2. **The primer and probe sequences for the qPCR assays.**

Gene target	Forward primer	Reverse primer	Probe
Shigella			
gtrI	AAATATGCCTCCATACAATTG	AGCATATGTATTAAACAATCAGCA	GCTGTTAGCAACATCCGGTTCAAC
gtrIc	ACCTTAGGTTCAAATGGGTTAC	GAAATAGCCGTCTCTCGAATA	TGTTTTCACATTTAGTATTCCAAC
gtrII	CAAACGACTCAGGAAATATGC	AATTCATAAATGCAACCATCCT	CTCCATGAGCGCAGACACTTTTG
gtrIV	TCTCACATGATGGCACATTA	CCTAAGATCAAATGTGTGTGTGA	TTTATACCCTGAAGGAAAATTTCAG
gtrV	TTATTAATGTCATCGTCCATCC	CCACTCCCAGATTACGG	GCAGGGTTGAACTTGAAAGAATACGAT
gtrX	AGCACCACATCAAAAATCTTC	CATACAATGATAAATACCAGTGAGCATT	TATATTTAATTTGCATGCCCGGGC
oac	GCATAAGAGCAACTGCTTTG	CGCGTAGTGGTGACTG	ACGGCAAGGCTTGTGGCA
wzx6	GAGCGATCATTTCAACTTCA	TACAACATGATTCGCGTTAATGT	CGGTAATTCTAACTATATTGGGCTTG
Rhs	CAGTGGCTGGCATATTTCGA	TGCTATACTCCAATCCTGTCTGTTTT	CTGACGCCGGAGTAGAT
pm	TGCCGCTAAAATCCTTCTGT	GCGTACGACGAAAGGAAAAA	AAGTTATTGATTCCGCCC
ipaH	CCTTTTCCGCGTTCCTTGA	CGGAATCCGGAGGTATTGC	CGCCTTTCCGATACCGTCTCTGCA
PhHV			
gB	GGGCGAATCACAGATTGAATC	GCGGTTCCAAACGTACCAA	TATGTGTCCGCCACCATCT

Results

Table S3. Number and type of samples by site among 2328 samples that were retested with the *Shigella* typing assays in the MAL-ED study.

Site	Total number of tested stools	Number of tested diarrhoeal stools	Number of tested non-diarrhoeal stools
**Bangladesh**	483	206 (0.43)	277 (0.57)
**Brazil**	51	9 (0.18)	42 (0.82)
**India**	342	90 (0.26)	252 (0.74)
**Nepal**	264	86 (0.33)	178 (0.67)
**Peru**	568	210 (0.37)	358 (0.63)
**Pakistan**	179	91 (0.51)	88 (0.49)
**South Africa**	112	7 (0.06)	105 (0.94)
**Tanzania**	443	19 (0.04)	424 (0.96)
**All**	2328	698 (0.30)	1630 (0.70)

Figure S1. Distribution of *ipaH* Cts by *Shigella* species detected at each site.

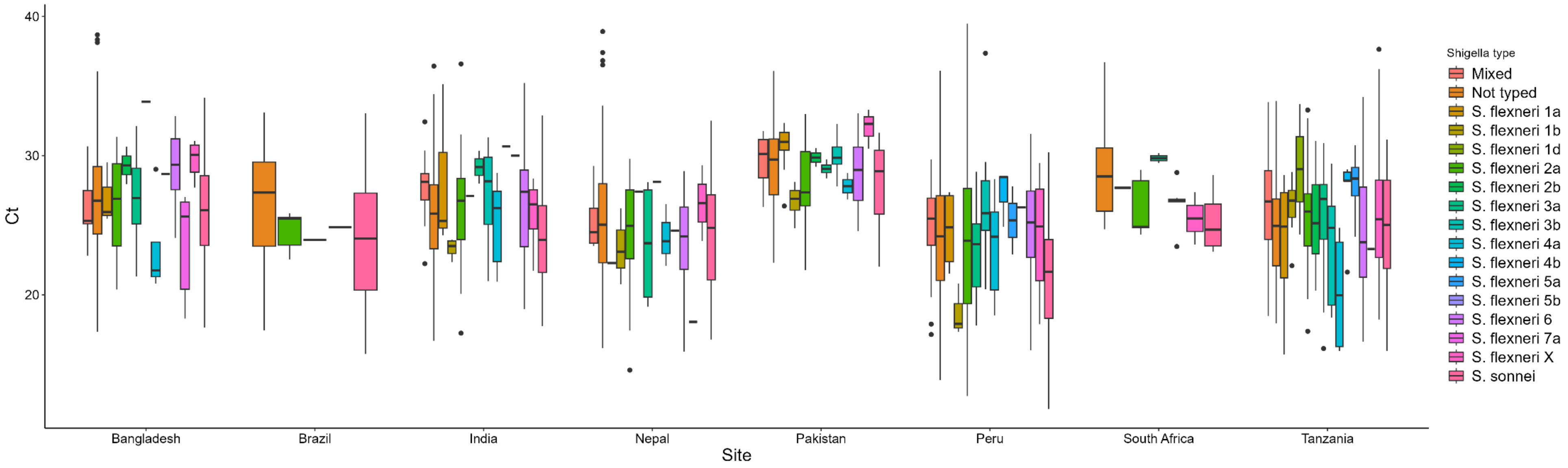

Note: outliers are defined as data points that fall outside 1.5 times the interquartile range from the first quartile or third quartile.

Table S4. ***S. sonnei*** **and** ***S. flexneri*** **serotype distribution across sites among 2295 samples that had detectable** ***ipaH*** **on retest with the** ***Shigella*** **typing assays in the MAL-ED study.**

	Site; N (%)								
Shigella **species/serotype**	**Bangladesh N=477**	**Brazil N=49**	**India N=312**	**Nepal N=257**	**Peru N=554**	**Pakistan N=172**	**South Africa N=64**	**Tanzania N=410**	**All N=2295**
S. flexneri	118 (24·7)	7 (14·3)	116 (37·2)	81 (31·5)	213 (38·4)	72 (41·9)	17 (27·4)	240 (58·5)	864 (37·7)
S. flexneri **1a**	4 (0·8)	0 (0)	7 (2·2)	2 (0·8)	5 (0·9)	15 (8·7)	0 (0)	7 (1·7)	40 (1·7)
S. flexneri **1b**	0 (0)	0 (0)	4 (1·3)	3 (1·2)	3 (0·5)	4 (2·3)	1 (1·6)	8 (2)	23 (1)
S. flexneri **1d**	0 (0)	0 (0)	0 (0)	0 (0)	1 (0·2)	0 (0)	0 (0)	2 (0·5)	3 (0·1)
S. flexneri **2a**	41 (8·6)	5 (10·2)	39 (12·5)	16 (6·2)	68 (12·3)	21 (12·2)	5 (8·1)	55 (13·4)	250 (10·9)
S. flexneri **2b**	2 (0·4)	1 (2)	1 (0·3)	1 (0·4)	0 (0)	3 (1·7)	0 (0)	17 (4·1)	25 (1·1)
S. flexneri **3a**	14 (2·9)	0 (0)	2 (0·6)	8 (3·1)	20 (3·6)	4 (2·3)	2 (3·2)	35 (8·5)	85 (3·7)
S. flexneri **3b**	1 (0·2)	0 (0)	6 (1·9)	1 (0·4)	10 (1·8)	7 (4·1)	0 (0)	10 (2·4)	35 (1·5)
S. flexneri **4a**	8 (1·7)	1 (2)	9 (2·9)	6 (2·3)	8 (1·4)	3 (1·7)	0 (0)	5 (1·2)	40 (1·7)
S. flexneri **4b**	1 (0·2)	0 (0)	1 (0·3)	1 (0·4)	6 (1·1)	0 (0)	0 (0)	7 (1·7)	16 (0·7)
S. flexneri **5a**	0 (0)	0 (0)	1 (0·3)	3 (1·2)	2 (0·4)	0 (0)	0 (0)	7 (1·7)	13 (0·6)
S. flexneri **5b**	0 (0)	0 (0)	1 (0·3)	0 (0)	1 (0·2)	0 (0)	0 (0)	1 (0·2)	3 (0·1)
S. flexneri **6**	42 (8·8)	0 (0)	49 (15·7)	37 (14·4)	80 (14·4)	16 (9·3)	7 (11·3)	65 (15·9)	296 (12·9)
S. flexneri **7a**	8 (1·7)	0 (0)	0 (0)	1 (0·4)	0 (0)	0 (0)	0 (0)	1 (0·2)	10 (0·4)
S. flexneri **X**	7 (1·5)	0 (0)	5 (1·6)	2 (0·8)	19 (3·4)	5 (2·9)	2 (3·2)	36 (8·8)	76 (3·3)
S. sonnei	76 (15·9)	15 (30·6)	62 (19·9)	66 (25·7)	58 (10·5)	30 (17·4)	5 (8·1)	54 (13·2)	366 (16)
**Mixed***	14 (2·9)	0 (0)	14 (4·5)	3 (1·2)	15 (2·7)	8 (4·7)	0 (0)	22 (5·4)	76 (3·3)
S. flexneri **2a, 3a, 6, or** ***S. sonnei***†	166 (34·8)	20 (40·8)	146 (46·8)	125 (48·6)	218 (39·4)	69 (40·1)	19 (30·6)	203 (49·5)	966 (42·1)
S. flexneri **1b, 2a, 3a, or** ***S. sonnei***†	129 (27)	20 (40·8)	106 (34)	93 (36·2)	144 (26)	58 (33·7)	13 (21)	151 (36·8)	714 (31·1)
S. flexneri **2a or** ***S. sonnei***†	115 (24·1)	20 (40·8)	100 (32·1)	82 (31·9)	123 (22·2)	50 (29·1)	10 (16·1)	109 (26·6)	609 (26·6)

*Mixed detections are counted for each individual species/serotype and for mixed.
†Combination of serotypes in leading vaccine candidate(s).

Figure S2. Risk ratio between *Shigella* quantity and diarrhoea by *Shigella* species in MAL-ED cohort.

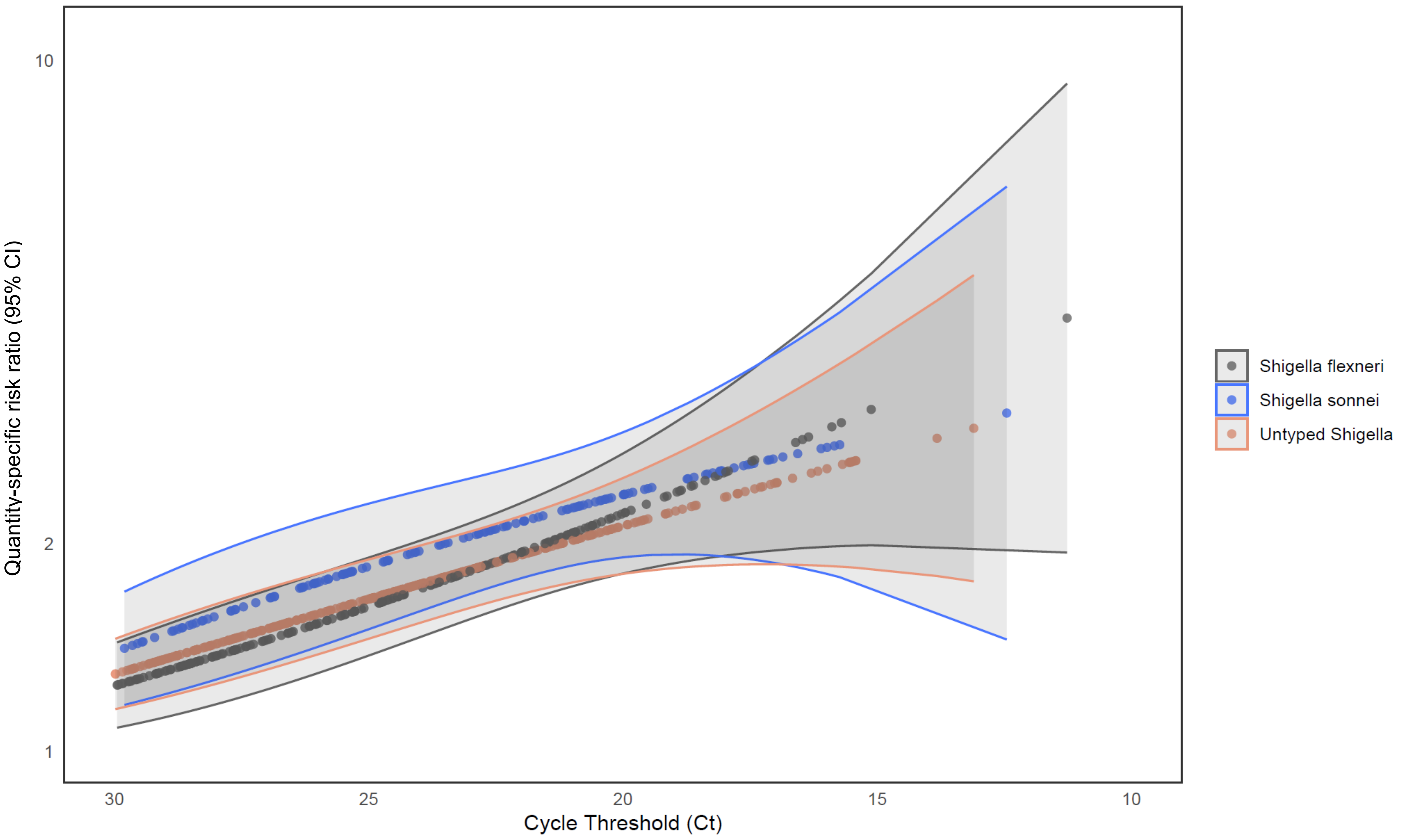

Association is the species- and quantity-specific risk ratio from a generalized linear mixed-effects Poisson regression model in which the outcome was diarrhoeal vs. non-diarrhoeal stool, and predictors were linear and quadratic terms for pathogen quantity (using the Ct value), child sex, TAC test batch, linear and quadratic terms for child age in three-month intervals, a random slope for each site, and a random intercept for each individual.

Table S5. Site-specific associations of subclinical *Shigella* infections with length-for-age z-score (LAZ) at two years of age and height-for-age z-score (HAZ) at five years of age in the MAL-ED cohort.

	Bangladesh	Brazil	India	Nepal	Peru	South Africa	Tanzania
**Effect of 1+ subclinical infection* on LAZ at 2 years†**							
S. flexneri	-0·31 (-0·55, -0·07)	-0·49 (-1·31, 0·34)	-0·07 (-0·29, 0·15)	-0·08 (-0·33, 0·17)	-0·16 (-0·36, 0·05)	-0·29 (-0·77, 0·20)	-0·14 (-0·38, 0·10)
S. sonnei	-0·08 (-0·38, 0·21)	0·28 (-0·44, 1·00)	0·02 (-0·25, 0·28)	0·09 (-0·19, 0·38)	-0·21 (-0·53, 0·1)	0·00 (-0·93, 0·93)	-0·29 (-0·58, 0·00)
**Type unknown**	-0·06 (-0·27, 0·15)	-0·05 (-0·47, 0·38)	0·13 (-0·08, 0·34)	0·30 (0·08, 0·52)	-0·12 (-0·33, 0·09)	-0·05 (-0·31, 0·22)	-0·23 (-0·46, 0·01)
**Effect of 1+ subclinical infection* on HAZ at 5 years†**							
S. flexneri	-0·15 (-0·42, 0·12)	-0·47 (-1·33, 0·39)	-0·21 (-0·43, 0·02)	-0·23 (-0·59, 0·13)	-0·28 (-0·52, -0·03)	-0·31 (-0·74, 0·13)	-0·23 (-0·48, 0·02)
S. sonnei	-0·06 (-0·38, 0·27)	-0·02 (-0·66, 0·62)	-0·03 (-0·30, 0·24)	0·05 (-0·31, 0·42)	-0·02 (-0·43, 0·39)	0·20 (-0·64, 1·03)	-0·15 (-0·46, 0·15)
**Type unknown**	-0·05 (-0·29, 0·18)	-0·22 (-0·64, 0·20)	0·13 (-0·08, 0·35)	0·43 (0·09, 0·76)	0·21 (-0·07, 0·49)	-0·19 (-0·43, 0·05)	-0·29 (-0·53, -0·05)

*Subclinical infections were defined as at least one detection with *ipaH* Ct<30 since typing assays were only conducted among samples with *ipaH* Ct<30.

†Adjusted for site, enrolment LAZ, sex, socioeconomic status, exclusive breastfeeding in the first six months, maternal height, and proportion of non-diarrhoeal stools positive for *Cryptosporidium*, *Campylobacter spp.*, *Giardia*, enteroaggregative *E. coli*, enterotoxigenic *E. coli*, typical enteropathogenic *E. coli*, atypical enteropathogenic *E. coli*, norovirus, adenovirus 40/41, astrovirus, sapovirus, and *Enterocytozoon bieneusi*.

Table S6. Association of *Shigella* species with myeloperoxidase concentration in the same stool sample.

	Adjusted myeloperoxidase concentration (ng/mL) difference (95% CI)		
	S. flexneri **(N=864)**	***S. sonnei*** **(N=365)**	***Shigella*** **of unknown type* (N=3546)**
Shigella **quantity**			
**Any**	1·58 (1·36, 1·84)	1·77 (1·38, 2·27)	1·72 (1·54, 1·72)
**1st tertile**	1·55 (1·20, 1·99)	1·57 (0·84, 2·94)	1·15 (1·06, 1·26)
**2nd tertile**	1·52 (1·25, 1·84)	1·78 (1·35, 2·36)	1·72 (1·51, 1·97)
**3rd tertile**	2·66 (1·63, 4·35)	2·12 (1·17, 3·86)	2·41 (1·86, 3·13)
**Per log increase in quantity**	1·12 (1·08, 1·16)	1·13 (1·07, 1·19)	1·13 (1·11, 1·15)

**Shigella* of unknown type detections were defined as at least one detection with *ipaH* Ct<30 since typing assays were only conducted among samples with *ipaH* Ct<30.

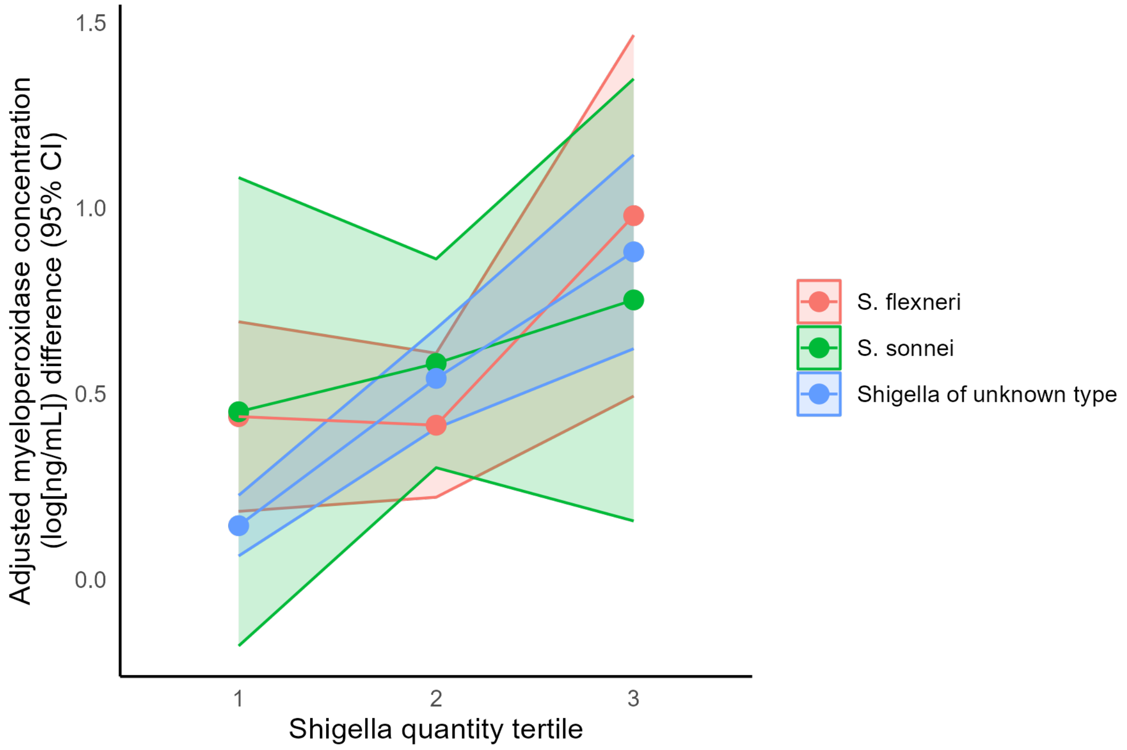

Figure S3. Dose response between quantity of specific *Shigella* species detected with myeloperoxidase concentration in the same stool sample.

Table S7. Median time to subsequent outcome (infection or diarrhoea) following a prior infection by *Shigella* species or serotype.

Shigella species/serotype	Outcome	Number of repeat infections or diarrhoea of the same type	Median time to subsequent infection or diarrhoea (days; IQR)
S. flexneri	Infection	277	62 (31, 181)
	Diarrhoea	43	77 (41, 175)
S. sonnei	Infection	56	47 (30, 109)
	Diarrhoea	7	40 (27, 103)
S. flexneri 2a	Infection	42	45 (31, 123)
	Diarrhoea	8	90 (36, 230)
S. flexneri 3a	Infection	11	30 (24, 72)
	Diarrhoea	1	44
S. flexneri 6	Infection	57	61 (31, 93)
	Diarrhoea	6	76 (47, 261)
S. flexneri X	Infection	6	34 (27, 90)
	Diarrhoea	0	--

Table S8. Estimated of protection against *Shigella* species/serotype specific subclinical infections and attributable diarrhoea due to 1 or more prior infections (asymptomatic or diarrhoea) from the same species/serotype in the MAL-ED cohort in the first (0-11 months) and second (12-23 months) years of life.

		Adjusted* hazard ratio (95% CI)		
Shigella species/serotype	Outcome	0-23 months	0-11 months	12-23 months
S. flexneri	Infection	2·11 (1·73, 2·57)	5.34 (3.14, 9.10)	1.91 (1.57, 2.31)
	Diarrhoea	1·65 (1·14, 2·38)	0.84 (0.11, 6.19)	1.71 (1.18, 2.47)
S· sonnei	Infection	2·14 (1·52, 3·02)	4.94 (2.23, 10.92)	1.98 (1.34, 2.91)
	Diarrhoea	0·57 (0·26, 1·25)	--	0.62 (0.28, 1.36)
S· flexneri 2a	Infection	3·07 (1·93, 4·90)	8.14 (2.38, 27.85)	2.79 (1.72, 4.54)
	Diarrhoea	2·03 (0·90, 4·56)	--	2.13 (0.94, 4.84)
S· flexneri 3a	Infection	3·82 (1·71, 8·49)	12.08 (1.51, 96.82)	3.64 (1.60, 8.28)
	Diarrhoea	2·25 (0·30, 16·99)	--	2.19 (0.28, 17.24)
S· flexneri 6	Infection	3·24 (2·32, 4·51)	9.33 (3.22, 27.00)	2.82 (1.98, 4.01)
	Diarrhoea	2·31 (0·90, 5·94)	10.26 (1.60, 65.79)	1.93 (0.71, 5.24)
S· flexneri X	Infection	1·71 (0·59, 5·00)	--	1.72 (0.58, 5.09)
	Diarrhoea	--	--	--

*Hazard ratios adjusted for site, socioeconomic status, sex, enrolment weight-for-age Z-score, maternal education, maternal height, crowding, and exclusive breastfeeding in first 6 months.

Table S9. Estimates of protection against *Shigella* species/serotype specific subclinical infections and attributable diarrhoea due to 1 or more prior infections (asymptomatic or diarrhoea) from the same species/serotype in the MAL-ED cohort, additionally adjusting for antibiotic use.

Shigella species/serotype	Outcome	Adjusted* hazard ratio (95% CI)	Adjusted† hazard ratio (95% CI)
S. flexneri	Infection	2·11 (1·73, 2·57)	2·11 (1·73, 2·58)
	Diarrhoea	1·65 (1·14, 2·38)	1·66 (1·15, 2·39)
S· sonnei	Infection	2·14 (1·52, 3·02)	2·16 (1·53, 3·04)
	Diarrhoea	0·57 (0·26, 1·25)	0·58 (0·27, 1·26)
S· flexneri 2a	Infection	3·07 (1·93, 4·90)	3·07 (1·92, 4·92)
	Diarrhoea	2·03 (0·90, 4·56)	2·04 (0·90, 4·62)
S· flexneri 3a	Infection	3·82 (1·71, 8·49)	3·81 (1·71, 8·48)
	Diarrhoea	2·25 (0·30, 16·99)	2·25 (0·30, 16·78)
S· flexneri 6	Infection	3·24 (2·32, 4·51)	3·26 (2·34, 4·54)
	Diarrhoea	2·31 (0·90, 5·94)	2·32 (0·90, 5·99)
S· flexneri X	Infection	1·71 (0·59, 5·00)	1·72 (0·59, 5·02)
	Diarrhoea	--	--

*Hazard ratios adjusted for site, socioeconomic status, sex, enrolment weight-for-age Z-score, maternal education, maternal height, crowding, and exclusive breastfeeding in first 6 months.

†Hazard ratios adjusted for the same variables, any antibiotic use and any macrolide/fluoroquinolone use in the 15 days before or after the first day of the prior infection.

Table S10. Estimates of protection against *Shigella* species/serotype specific attributable diarrhoea due to 1 or more prior diarrhoea episodes from the same species/serotype in the MAL-ED cohort.

Shigella species/serotype†	Unadjusted hazard ratio (95% CI)	Adjusted* hazard ratio (95% CI)
S. flexneri	1.47 (1.03, 2.12)	1.45 (1.01, 2.09)
S. sonnei	0.57 (0.26, 1.23)	0.53 (0.25, 1.16)
S. flexneri 2a	1.93 (0.89, 4.17)	1.86 (0.84, 4.13)

*Hazard ratios adjusted for site, socioeconomic status, sex, enrolment weight-for-age Z-score, maternal education, maternal height, crowding, and exclusive breastfeeding in first 6 months.

†Hazard ratios could not be estimated for other *S. flexneri* serotypes due to small numbers.

Table S11. Expressions of cross protection from *S. flexneri* 2a and *S. flexneri* 3a against other *S. flexneri* serotypes and *S. sonnei*.[2]

	Expectation of cross protection from *S. flexneri 2a*	No expectation of cross protection from *S. flexneri 2a*	Expectation of cross protection from *S. flexneri 3a*	No expectation of cross protection from *S. flexneri 3a*
***S. flexneri* 1a**	X			X
***S. flexneri* 1b**		X		X
***S. flexneri* 1d**				
***S. flexneri* 2a**	Direct protection			
***S. flexneri* 2b**	X		X	
***S. flexneri* 3a**		X	Direct protection	
***S. flexneri* 3b**	X		X	
***S. flexneri* 4a**	X			X
***S. flexneri* 4b**		X	X	
***S. flexneri* 5a**	X			X
***S. flexneri* 5b**		X	X	
***S. flexneri* 6**		X		X
***S. flexneri* 7a**		X		X
***S. flexneri* X**		X	X	
S. sonnei		X		X

References

1 Liu J, Pholwat S, Zhang J, et al. Evaluation of Molecular Serotyping Assays for Shigella flexneri Directly on Stool Samples. *J Clin Microbiol*. **2021**; 59(2).

2 Levine MM, Kotloff KL, Barry EM, Pasetti MF, Sztein MB. Clinical trials of Shigella vaccines: two steps forward and one step back on a long, hard road. *Nat Rev Microbiol*. **2007**; 5(7):540–553.
